# Ringlike late gadolinium enhancement provides incremental prognostic value in non-classical arrhythmogenic cardiomyopathy

**DOI:** 10.1186/s12968-023-00986-1

**Published:** 2023-11-30

**Authors:** Yuelong Yang, Xiaoyu Wei, Guanyu Lu, Jiajun Xie, Zekun Tan, Zhicheng Du, Weitao Ye, Huanwen Xu, Xiaodan Li, Entao Liu, Qianhuan Zhang, Yang Liu, Jinglei Li, Hui Liu

**Affiliations:** 1 Department of Radiology, Guangdong Provincial People’s Hospital (Guangdong Academy of Medical Sciences), Southern Medical University, Guangzhou, 510080 China; 2 Department of Radiology, Sun Yat-Sen Memorial Hospital, Sun Yat-Sen University, Guangzhou, 510120 China; 3 Department of Interventional Diagnosis and Therapy, Beijing Anzhen Hospital, Capital Medical University, Beijing, 100029 China; 4 Department of Radiology, Guangzhou First People’s Hospital, School of Medicine, South China University of Technology, Guangzhou, 510080 China; 5 Guangdong Cardiovascular Institute, Guangdong Provincial People’s Hospital (Guangdong Academy of Medical Sciences), Southern Medical University, Guangzhou, 510080 China; 6 Department of Medical Statistics, School of Public Health, Sun Yat-Sen University, Guangzhou, 510080 China; 7 WeiLun PET Center, Department of Nuclear Medicine, Guangdong Provincial People’s Hospital (Guangdong Academy of Medical Sciences), Southern Medical University, Guangzhou, 510080 China; 8 Guangdong Provincial Key Laboratory of Artificial Intelligence in Medical Image Analysis and Application, Guangdong Provincial People’s Hospital, Guangdong Academy of Medical Sciences, Guangzhou, 510080 China

**Keywords:** Cardiac magnetic resonance, Ringlike late gadolinium enhancement, Arrhythmogenic cardiomyopathy, Ventricular arrhythmia

## Abstract

**Background:**

The 2019 arrhythmogenic right ventricular cardiomyopathy (ARVC) risk model has proved insufficient in the capability of predicting ventricular arrhythmia (VA) risk in non-classical arrhythmogenic cardiomyopathy (ACM). Furthermore, the prognostic value of ringlike late gadolinium enhancement (LGE) of the left ventricle in non-classical ACM remains unknown. We aimed to assess the incremental value of ringlike LGE over the 2019 ARVC risk model in predicting sustained VA in patients with non-classical ACM.

**Methods:**

In this retrospective study, consecutive patients with non-classical ACM who underwent CMR from January 2011 to January 2022 were included. The pattern of LGE was categorized as no, non-ringlike, and ringlike LGE. The primary outcome was defined as the occurrence of sustained VA. Univariable and multivariable Cox regression analysis was used to evaluate the impact of LGE patterns on sustained VA and area under curve (AUC) was calculated for the incremental value of ringlike LGE.

**Results:**

A total of 73 patients were collected in the final cohort (mean age, 39.3 ± 14.4 years, 51 male), of whom 10 (13.7%) had no LGE, 33 (45.2%) had non-ringlike LGE, and 30 (41.1%) had ringlike LGE. There was no statistically significant difference in the 5-year risk score among the three groups (*P* = 0.190). During a median follow-up of 34 (13–56) months, 34 (46.6%) patients experienced sustained VA, including 1 (10.0%), 13 (39.4%) and 20 (66.7%) of patients with no, non-ringlike and ringlike LGE, respectively. After multivariable adjustment, ringlike LGE remained independently associated with the presence of sustained VA (adjusted hazard ratio: 6.91, 95% confidence intervals: 1.89–54.60; *P* = 0.036). Adding ringlike LGE to the 2019 ARVC risk model showed significantly incremental prognostic value for sustained VA (AUC: 0.80 vs. 0.67; *P* = 0.024).

**Conclusion:**

Ringlike LGE provides independent and incremental prognostic value over the 2019 ARVC risk model in patients with non-classical ACM.

**Supplementary Information:**

The online version contains supplementary material available at 10.1186/s12968-023-00986-1.

## Introduction

Arrhythmogenic cardiomyopathy (ACM), an inherited non-ischemic cardiomyopathy characterized predominantly by fibrofatty myocardial replacement, is one of the leading causes of ventricular arrhythmias (VA) and sudden cardiac death (SCD) in young people and athletes [1]. Traditionally, the right ventricle is the first and predominant site of involvement in arrhythmogenic right ventricular cardiomyopathy (ARVC) [also known as right dominant ACM or classic ACM (R-ACM)] [2]. However, with the widespread application of genome sequencing and cardiac magnetic resonance (CMR) technologies, there has been an increasing number of diagnoses of non-classical ACM referring to biventricular or left dominant disease forms which were reported in almost 50% of cases of ACM [3].

So far, with no curative treatment options for ACM, accurate arrhythmic risk stratification and VA/SCD prevention are essential for patient management. The placement of an implantable cardioverter-defibrillator (ICD) is an effective treatment to prevent SCD. Although several expert consensus documents establishing the flow diagram algorithms for ICD implantation have been provided [4–6], the algorithms overlooking the interactive effects of multiple risk factors may limit their accuracy in a real-world environment [7]. Recently, a 2019 novel ARVC risk model for VA risk stratification in patients with ARVC was proposed by Cadrin-Tourigny et al. [8]. The risk model showed a good performance for the estimation of VA risk in patients with classic ACM which was validated in external independent cohorts, but seemed to underestimate the risk of VA in non-classical ACM with left ventricular (LV) involvement [9].

The extent and pattern of late gadolinium enhancement (LGE) were useful to further improve risk stratification for patients, and a greater LGE extent and a multi-focal LGE pattern were the independent predictors of adverse cardiac events [10, 11]. Prior studies have shown that the presence of LGE in the LV was associated with adverse outcomes, including malignant VA and SCD in patients with ACM [12]. Interestingly, a specific LV LGE phenotype characterized by a ringlike pattern has proved to be independently associated with VA in dilated cardiomyopathy (DCM) [13]. However, the relationship between ringlike LGE and VA has not been determined in non-classical ACM.

In this study, we thus assumed that LV imaging biomarkers, especially in terms of the location and distribution of LV LGE, may provide additional predictive information for non-classical ACM. The aim of the present study was to (1) evaluate the ability of ringlike LGE to predict the occurrence of sustained VA and (2) assess the incremental value of ringlike LGE over the 2019 ARVC risk model in patients with non-classical ACM.

## Materials and methods

### Study population

This study was approved by the institutional review board, and informed consent was waived owing to the study’s retrospective nature. Depending on the disease phenotypes and ventricle involvement, patients were classified as having classical ACM (R-ACM) and non-classical ACM, the latter of which was further classified into biventricular ACM (Bi-ACM) and left dominant ACM (LD-ACM). The diagnosis of R-ACM and non-classical ACM were based on the “Padua Criteria” proposed in recent years (Additional file 1: Diagnostic criteria) [14], R-ACM was considered in patients who predominantly showed right ventricle (RV) involvement, without morpho-functional and/or structural LV abnormalities; Bi-ACM was diagnosed when patients meeting ≥ 1 morpho-functional and/or structural abnormalities of both the RV and LV (i.e., patients fulfilling both RV and LV phenotypic criteria); LD-ACM was diagnosed in patients who showed structural LV abnormalities (with or without morpho-functional alterations), with the demonstration of an ACM-causing gene-mutation, in the absence of RV abnormalities.

**Fig. 1:**
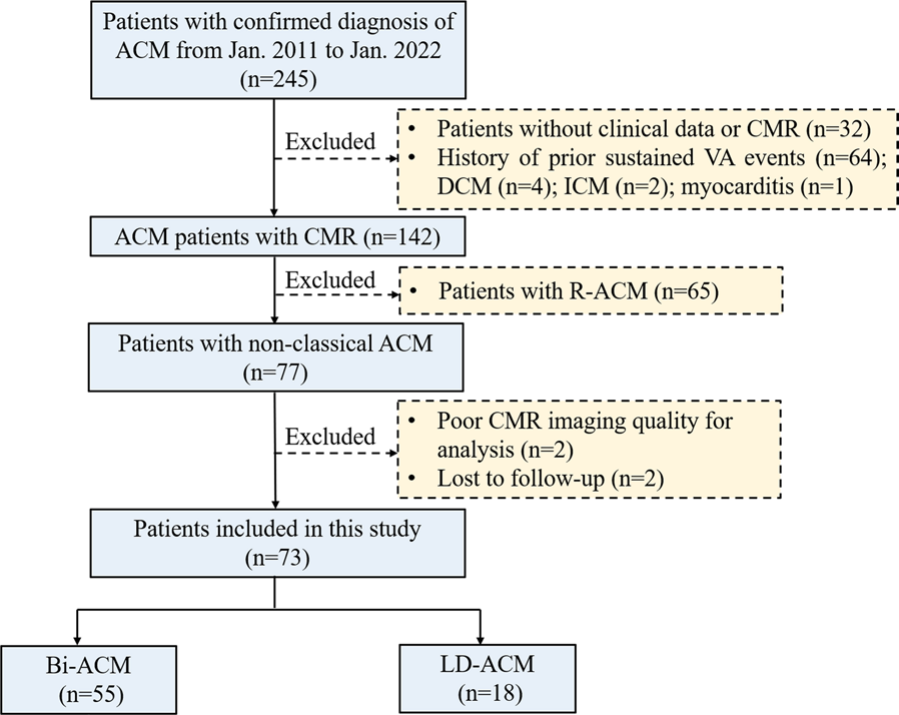
Flowchart of patient inclusion and exclusion criteria.  *ACM* arrhythmogenic cardiomyopathy, *CMR* cardiac magnetic resonance, *VA* ventricular arrhythmia, *DCM* dilated cardiomyopathy, *ICM* ischemic cardiomyopathy, *R-ACM* classical ACM, *Bi-ACM* biventricular ACM, *LD-ACM* left dominant ACM

This study included 245 consecutive patients with a diagnosis of ACM from January 2011 to January 2022 at our institution, all patients were retrospectively assessed and diagnosed using the “Padua Criteria”, the diagnosis of ACM is made by comprehensive evaluation of CMR images and clinical information by radiologists and cardiologists with extensive experience. The exclusion criteria included patients without clinical data or CMR, history of prior sustained VA events due to the 2019 ARVC risk model built to predict the first sustained VA, DCM, ischemic cardiomyopathy, myocarditis, disease phenotype classified as R-ACM, and poor CMR imaging quality for analysis. Therefore, the final study population included 73 patients: 55 with Bi-ACM and 18 with LD-ACM. Figure 1 shows the flowchart for patient inclusion and exclusion criteria.

### CMR protocol

All CMR images were obtained using a clinical 1.5T scanner (Achieva, Philips Healthcare) with 8 coil elements (Jan 2011–Sep 2014) and a 3T scanner (Ingenia, Philips Healthcare) with 32 coil elements (Oct 2014–Jan 2022) according to the recommended CMR protocol for ACM [15, 16]. The study mainly includes (1) T2-weighted imaging; (2) cine imaging; and (3) LGE imaging, the detailed protocol and parameters of sequences are shown in additional file (Additional file 1: CMR Protocol).

### CMR analysis

All the CMR image analyses were performed with a commercially available software (QMass software version 8.1, Medis, Leiden, The Netherlands) by experienced cardiologists (YL.Y. with more than 10 years of experience). The detailed process and parameters for cardiac structural and functional evaluation are shown in additional file (Additional file 1: CMR Analysis).

Areas of LGE were defined as myocardium with a signal intensity greater than 5 standard deviations (SD) above the mean signal intensity of remote reference myocardium [17]. Ringlike LGE pattern was identified if there were full involvement of at least three contiguous segments with LGE at the subepicardial or midmyocardial layer in the same short-axis slice (Additional file 1: Fig. S1) [18]. According to the presence and patterns of LV LGE, patients were classified into 3 groups: group I with no evidence of LGE, group II characterized by the presence of non-ringlike LGE pattern, and group III characterized by the presence of ringlike LGE pattern.

The method investigating the interobserver reproducibility in LGE pattern classification was shown in additional file (Additional file 1: CMR Analysis).

### Study outcomes

The primary outcome of this study was the first sustained VA following a definite ACM diagnosis. As adopted by previous studies [8], sustained VA was defined as a composite of sustained ventricular tachycardia (VT), ventricular fibrillation/flutter (VF), SCD, and appropriate ICD intervention. Sustained VT was defined as a tachycardia lasting ≥ 30 s at a rate ≥ 100 bpm or requiring intervention for termination due to haemodynamic compromise. SCD was defined as unexpected death within 1 h of the onset of cardiac symptoms or unwitnessed death such as during sleep or unexpected death within 24 h of last being seen alive. Appropriate ICD intervention was considered to ICD shock therapy or anti-tachycardia pacing therapy due to life-threatening arrhythmias (such as VF or sustained VT).

### Statistical analysis

Continuous variables were expressed as mean ± standard deviation or as median (interquartile range), which were compared between multiple groups using a one-way analysis of variance (ANOVA) test or a Kruskal-Wallis test, respectively. Categorical variables were presented as absolute numbers and percentages and compared using the χ^2^ test or Fisher’s exact test.

Univariable and multivariable Cox regression analysis was used to evaluate the potential predictors of VA. Variables with a *P* < 0.05 in univariable analyses should be included in the multivariable analysis. However, for variables with significant multicollinearity (defined as a correlation coefficient r > 0.70), only 1 independent variable with the lowest *P* value in the univariable analysis was included in the multivariable analysis. Considering the strong correlation (r = − 0.71; *P* < 0.001) between left ventricle ejection fraction (LVEF) and LV end-systolic volume index (ESVi) in the univariable analysis, only LVEF was introduced in the multivariable analysis to avoid overfitting. Kaplan-Meier survival curves were established to plot the time-to-event outcomes, which were compared using the log-rank test.

The ACM risk score was calculated using an online calculator at www.arvcrisk.com based on the 2019 ARVC risk model predicting the risk of sustained VA, which includes male sex, age, recent cardiac syncope, prior non-sustained ventricular tachycardia, 24-h premature ventricular contraction count, number of leads with T-wave inversion, and RVEF [8]. To evaluate the incremental value of LVEF, LGE extent and ringlike LGE, the procedure was as follows: First, we separately added them to the 2019 ARVC risk model (i.e. the 2019 ARVC risk model; the 2019 ARVC risk model + LVEF; the 2019 ARVC risk model + LGE extent; the 2019 ARVC risk model + ringlike LGE). Second, logistic regression was used to calculate the predicted probabilities of the multivariable models on sustained VA. Finally, the receiver-operating characteristic curve analysis with the DeLong method was performed to calculate and compare the area under curve (AUC) of different models with predicted probabilities.

Two-sided *P* < 0.05 were considered statistically significant. Statistical analyses were performed with SPSS version 24.0 (Statistical Package for the Social Sciences, International Business Machines, Inc., Armonk, New York, USA) and GraphPad Prism 6.0 (Graph-Pad Software, San Diego, California, USA).

## Results

### Study population characteristics

**Fig. 2:**
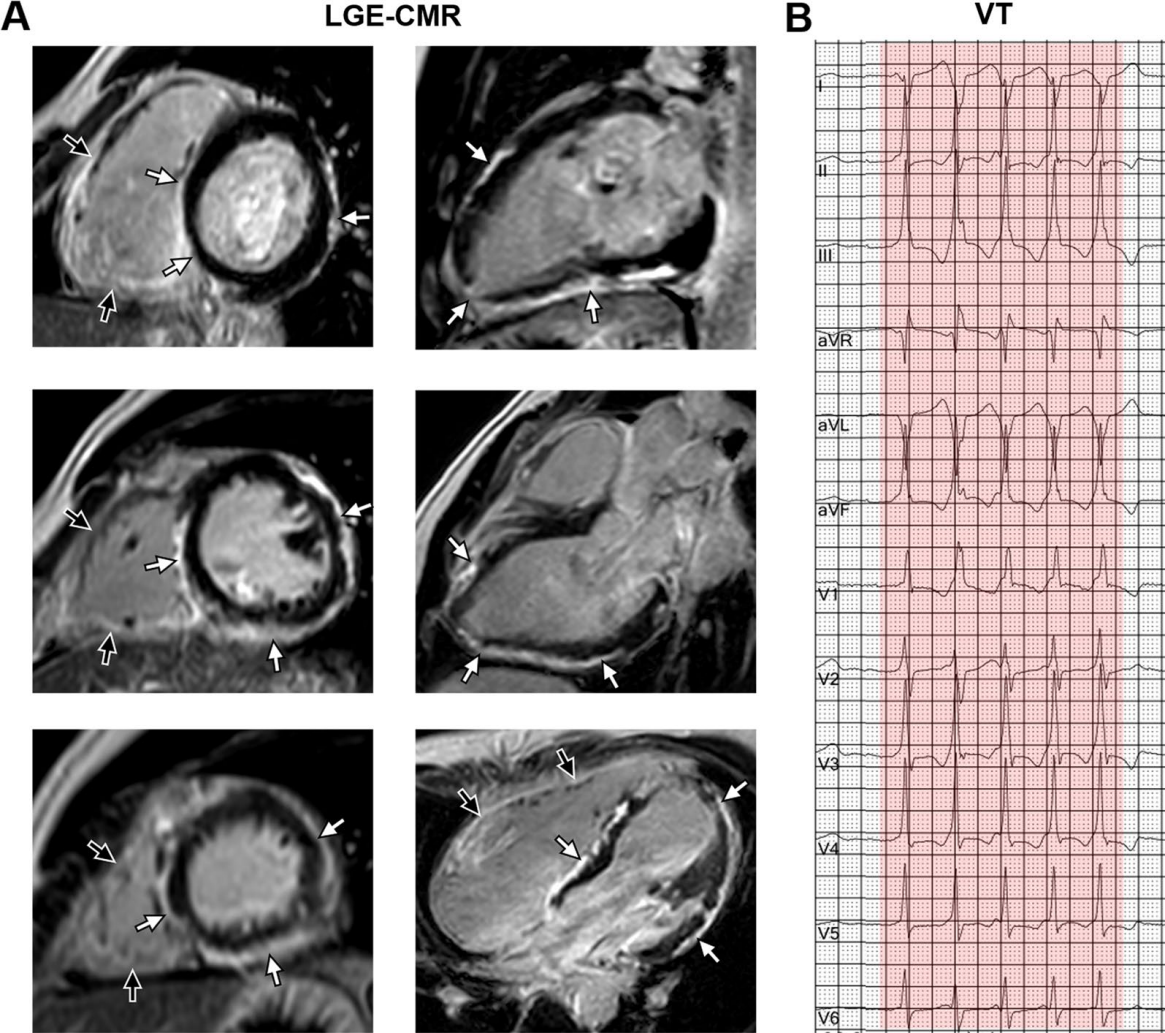
Ringlike LGE and corresponding electrocardiograph. A 25-year-old man had palpitation for 4 years with genetically diagnosed Bi-ACM (DSP mutation). **A** LGE-CMR images showed the extensive subepicardial fibrosis with a ringlike pattern involving the LV free wall and septum in both the short axis (left panel: from base to apex) and the long axis view (right panel: from 2 chamber to 4 chamber) (white arrowheads). Meanwhile, extensive LGE was also seen in the right ventricular wall (black arrowheads). **B** The 12-lead electrocardiograph showed monomorphic VT. *LGE* late gadolinium enhancement, *Bi-ACM* biventricular arrhythmogenic cardiomyopathy, *DSP* desmoplakin, *CMR* cardiac magnetic resonance, *LV* left ventricular, *VT* ventricular tachycardia

**Fig. 3:**
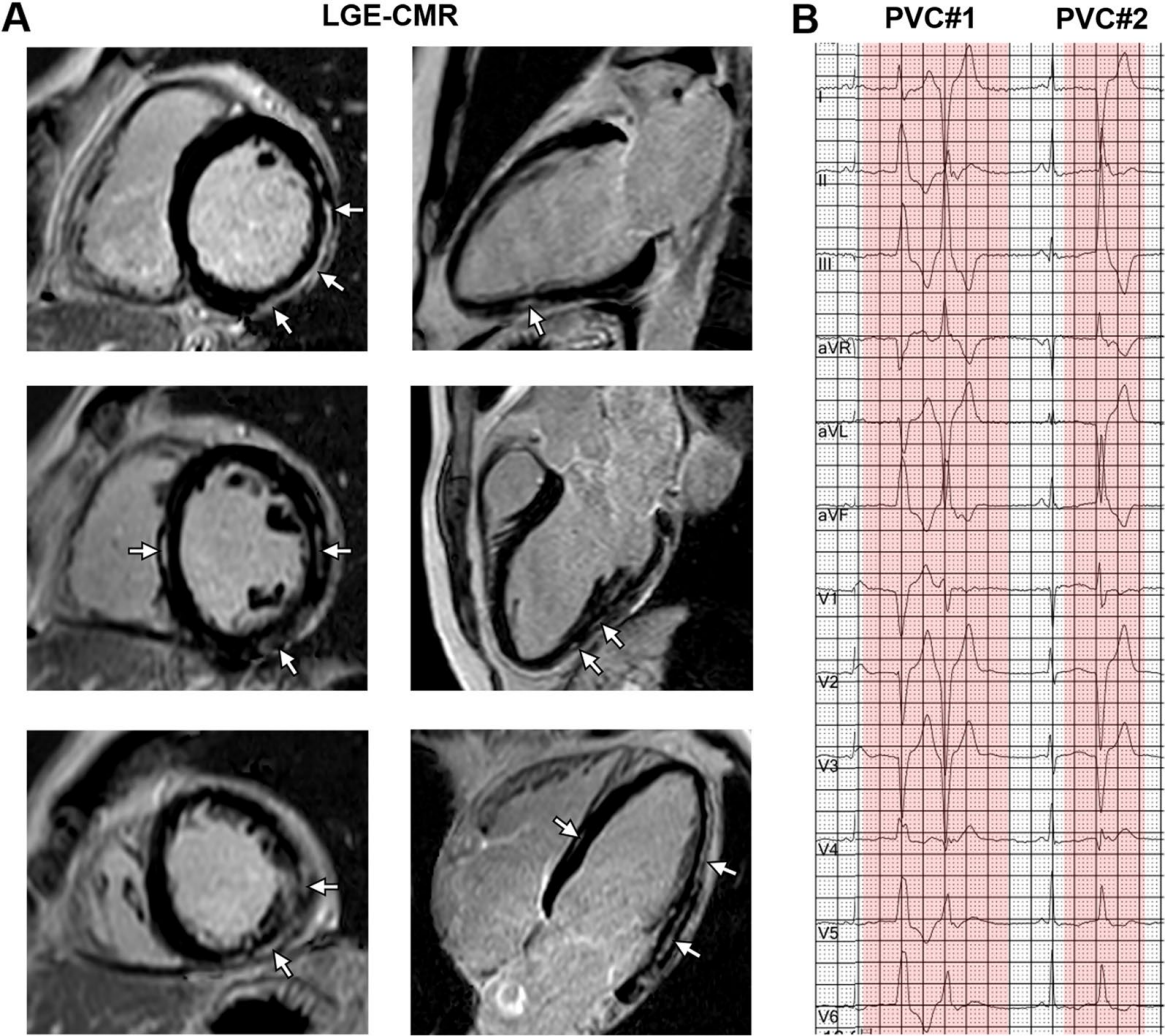
Non-ringlike LGE and corresponding electrocardiograph. A 17-year-old woman had a prior history of syncope for 1 year with genetically diagnosed LD- ACM (PKP2 mutation). **A** LGE-CMR images showed the multifocal subepicardial fibrosis with a non-ringlike pattern involving the LV free wall and septum in both the short axis (left panel: from base to apex) and the long axis view (right panel: from 2 chamber to 4 chamber) (white arrowheads). **B** The 12-lead electrocardiograph showed multifocal PVC. *LGE* late gadolinium enhancement, *LD-ACM* left dominant arrhythmogenic cardiomyopathy, *PKP2* plakophilin-2, *CMR* cardiac magnetic resonance, *LV* left ventricular, *PVC* premature ventricular contraction

The baseline clinical characteristics of the study population according to the patterns of LV LGE are shown in Table 1. In total, 73 non-classical ACM patients [mean age, 39.3 ± 14.4 years; 51 (69.9%) male] without prior sustained VA were included in the final analysis. Of which 63 (86.3%) patients had evidence of LV LGE, with 30 of 73 (41.1%) patients demonstrating a ringlike pattern (Fig. 2) and 33 of 73 (45.2%) a non-ringlike pattern (Fig. 3) of LGE. The remaining 10 of 73 (13.7%) patients had no LGE. Classification of the patterns of LGE showed excellent interobserver reproducibility (Kappa = 0.91, *P* < 0.001) (Additional file 1: Table S1). Compared to patients without LGE and non-ringlike LGE, patients with ringlike LGE did not show significant difference in the 5-year ARVC risk score for the presence of VA (15.9 ± 8.0 vs. 18.8 ± 7.7 vs. 20.8 ± 8.6%; *P* = 0.19). Thirty-two patients of 73 (43.8%) underwent genetic testing: PKP2 [15 of 73 (20.5%)] was the most frequently mutated gene compared with other genes. The second most common gene was DSP [8 of 73 (11.0%)], and most of them [6 of 8 (75.0%)] occurred in the ringlike LGE group.

**Table 1:** Baseline clinical characteristics according to the patterns of LV LGE

Variables	All patients (n = 73)	No LGE (n = 10)	Non-ringlike LGE (n = 33)	Ringlike LGE (n = 30)	*P-*Value
Clinical characteristics					
Age (years)	39.3 ± 14.4	30.6 ± 8.4	37.2 ± 14.9	44.5 ± 13.7	**0.017**
Male, n (%)	51(69.9)	4(40.0)	25(75.8)	22(73.3)	0.10
BSA (m^2^)	1.7 ± 0.2	1.7 ± 0.2	1.7 ± 0.1	1.6 ± 0.3	0.20
Systemic hypertension, n (%)	13(17.8)	2(20.0)	6(18.2)	5(16.7)	0.97
Diabetes, n (%)	7(9.6)	1(10.0)	2(6.1)	4(13.3)	0.61
History of syncope, n (%)	15(20.5)	1(10.0)	3(9.1)	11(36.7)	**0.017**
Family history of CAD, n (%)	5(6.8)	1(10.0)	2(6.1)	2(6.7)	0.92
Proband, n (%)	39(53.4)	4(40.0)	14(42.4)	21(70.0)	0.06
NYHA III–IV, n (%)	20(27.4)	0	6(18.2)	14(46.7)	**0.002**
NSVT, n (%)	41(56.2)	2(20.0)	21(63.6)	18(60.0)	0.051
24-h PVC count (≥ 1000), n (%)	49(67.1)	5(50.0)	23(69.7)	21(70.0)	0.46
Inverted T-wave, n	2(0–4)	3(0–4)	1(0–4)	3(0–5)	0.17
Genotype (n = 32), n (%)	32(43.8)	3(30.0)	13(39.4)	16(53.3)	0.36
PKP2	15(20.5)	2(20.0)	7(21.2)	6(20.0)	
DSP	8(11.0)	0	2(6.1)	6(20.0)	
DSG2	4 (5.5)	1(10.0)	1(3.0)	2(6.7)	
DSC2	2(2.7)	0	1(3.0)	1(3.3)	
Other	3(4.1)	0	2(6.1)	1(3.3)	
Therapy, n (%)					
Beta-blockers	59(80.8)	8(80.0)	26(78.8)	25(83.3)	0.90
ACE inhibitors	31(42.5)	3(30.0)	14(42.4)	14(46.7)	0.65
Antiarrhythmic drug	38(52.1)	5(50.0)	19(57.6)	14(46.7)	0.68
Diuretic agent	19(26.0)	3(30.0)	8(24.2)	8(26.7)	0.93
ICD	27(37.0)	2(20.0)	12(36.4)	13(43.3)	0.39
5-yr ARVC risk score, (%)	18.9 ± 8.2	15.9 ± 8.0	18.8 ± 7.7	20.8 ± 8.6	0.19
Sustained VA, n (%)	34(46.6)	1(10.0)	13(39.4)	20(66.7)	**0.003**

Continuous variables are presented as mean ± standard deviation or median (interquartile range) and discrete variables as n (%). Values in bold indicate P < 0.05

*LV* left ventricular, *LGE* late gadolinium enhancement, *BSA* body surface area, *CAD* coronary artery disease, *NYHA* New York Heart Association, *NSVT* non-sustained ventricular tachycardia, *PVC* premature ventricular complex, *PKP2* plakophilin-2, *DSP* desmoplakin, *DSG2* desmoglein-2, *DSC2* desmocollin-2, *ACE* angiotensin-converting enzyme, *ICD* implantable cardioverter-defibrillator, *ARVC* arrhythmogenic right ventricular cardiomyopathy, *VA* ventricular arrhythmia

### CMR characteristics

The CMR characteristics of the study population according to the patterns of LV LGE are shown in Table 2. Compared with the no LGE and non-ringlike LGE groups, the ringlike LGE group had a higher LV ESVi (64 ± 15 vs. 52 ± 18 vs. 49 ± 17 mL/m^2^; *P* = 0.002) and lower LVEF (41 ± 7.3 vs. 53 ± 8.4 vs. 45 ± 7.0%; *P* < 0.001), no significant difference was detected in the RV volume and function among the 3 groups. The extent of LV LGE was higher in the ringlike LGE group compared with the non-ringlike LGE group (25 [16–32] vs.18 [12–22] %; *P* < 0.001).

**Table 2:** CMR characteristics according to the patterns of LV LGE

Variables		All patients (n = 73)	No LGE (n = 10)	Non-ringlike LGE (n = 33)		Ringlike LGE (n = 30)		*P-*Value		
LV EDVi (mL/m^2^)		97 ± 16	93 ± 13	94 ± 17		101 ± 15		0.14		
LV ESVi (mL/m^2^)		55 ± 18	52 ± 18	49 ± 17		64 ± 15		**0.002**		
LV MI (g/m^2^)		67 ± 13	59 ± 8.6	67 ± 13		70 ± 12		0.054		
LV EF (%)		45 ± 8.2	53 ± 8.4	45 ± 7.0		41 ± 7.3		**< 0.001**		
LV RWMA, n (%)		27(37.0)	2(20.0)	16(48.5)		9(30.0)		0.20		
LV fat infiltration, n (%)		31(42.5)	4(40.0)	12(36.4)		15(50.0)		0.55		
LV LGE extent (%)		19(11–26)	–	18(12–22)		25(16–32)		**< 0.001**		
LV LGE distribution, n (%)										
Anterior wall		37(50.7)	–	17(51.5)		20(66.7)		0.22		
Septum		45(61.6)	–	21(63.6)		24(80.0)		0.15		
Inferior wall		51(69.9)	–	23(69.7)		28(93.3)		**0.039**		
Lateral wall		56(76.7)	–	26(78.8)		30(100.0)		**0.011**		
LV LGE location, n (%)										
Subepicardial		39(53.4)	–	14(42.4)		25(83.3)		**0.001**		
Middle wall		23(31.5)	–	18(54.5)		5(16.7)		**0.002**		
Subendocardial		4(5.5)	–	4(12.1)		0		0.15		
RV EDVi (mL/m^2^)		100 ± 27	92 ± 26	101 ± 30		100 ± 22		0.60		
RV ESVi (mL/m^2^)		48 ± 15	43 ± 14	48 ± 18		49 ± 13		0.59		
RV EF (%)		52 ± 5.5	53 ± 5.9	53 ± 5.8		52 ± 5.3		0.64		
RV RWMA, n (%)		31(42.5)	4(40.0)	11(33.3)		16(53.3)		0.27		
RV fat infiltration, n (%)		29(39.7)	2(20.0)	11(33.3)		16(53.3)		0.12		
RV LGE, n (%)		36(49.3)	3(30.3)	15(45.5)		18(60.0)		0.23		

Continuous variables are presented as mean ± standard deviation or median (interquartile range) and discrete variables as n (%). Values in bold indicate P < 0.05

*LV* left ventricular, *CMR* cardiac magnetic resonance, *LGE* late gadolinium enhancement, *EDVi* end diastolic volume index, *ESVi* end systolic volume index, *MI* mass index, *EF* ejection fraction, *RWMA* regional wall motion abnormalities, *RV* right ventricular

### Distribution and location of LGE

**Fig. 4:**
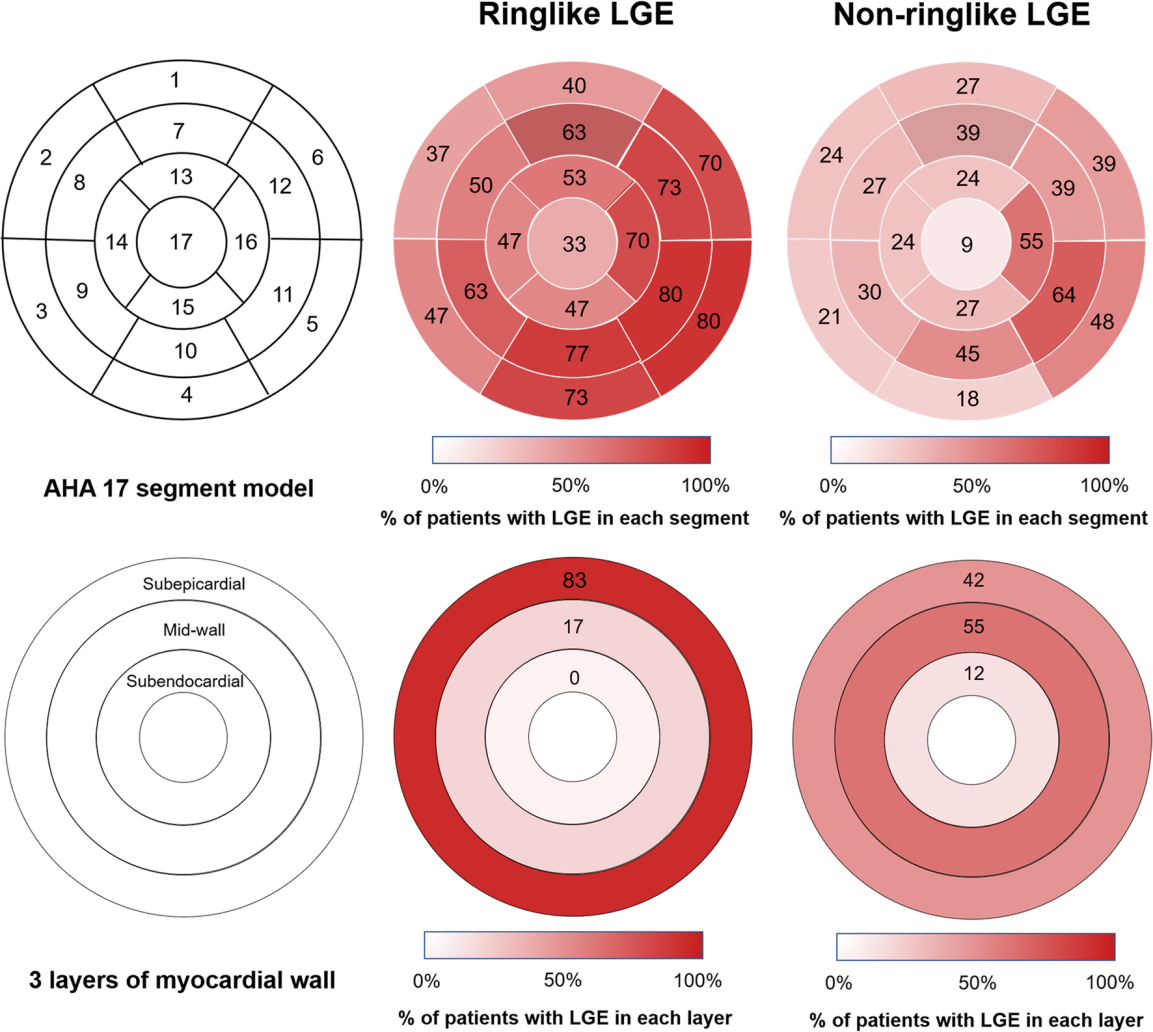
Distribution and location of different LGE patterns in the left ventricle. The distribution of LGE in patients with ringlike LGE was located predominantly at the lateral and inferior walls in the subepicardial layer. The distribution of LGE in patients with non-ringlike LGE was located predominantly at the lateral wall in mid-wall layer. *LGE* late gadolinium enhancement

The overall distribution of LGE was different between the non-ringlike LGE and the ringlike LGE groups (Fig. 4), with the ringlike LGE group having a predominant inferior [28 of 30 (93.3%)] and lateral [30 of 30 (100.0%)] wall involvement versus 23 of 33 (69.7%) and 26 of 33 (78.8%) in the non-ringlike LGE group, respectively (*P* = 0.039 and *P* = 0.011, respectively). Septal involvement was observed in 24 of 30 (80.0%) patients in the ringlike LGE group versus 21 of 33 (63.6%) in the non-ringlike LGE group (*P* = 0.150). For LGE location in the three layers of the myocardial wall, the majority of LGE location [25 of 30 (83.3%)] was observed in the subepicardial layer in the ringlike LGE group and a few [5 of 30 (16.7%)] in the middle wall in comparison with 14 of 33 (42.4%) and 18 of 33 (54.5%) of the cases in the non-ringlike LGE group (*P* = 0.001 and *P* = 0.002, respectively). None of the patient in the ringlike LGE group had the evidence of subendocardial layer of LGE compared to 4 of 33 (12.1%) in the non-ringlike LGE group (*P* = 0.150).

### Outcomes

**Fig. 5:**
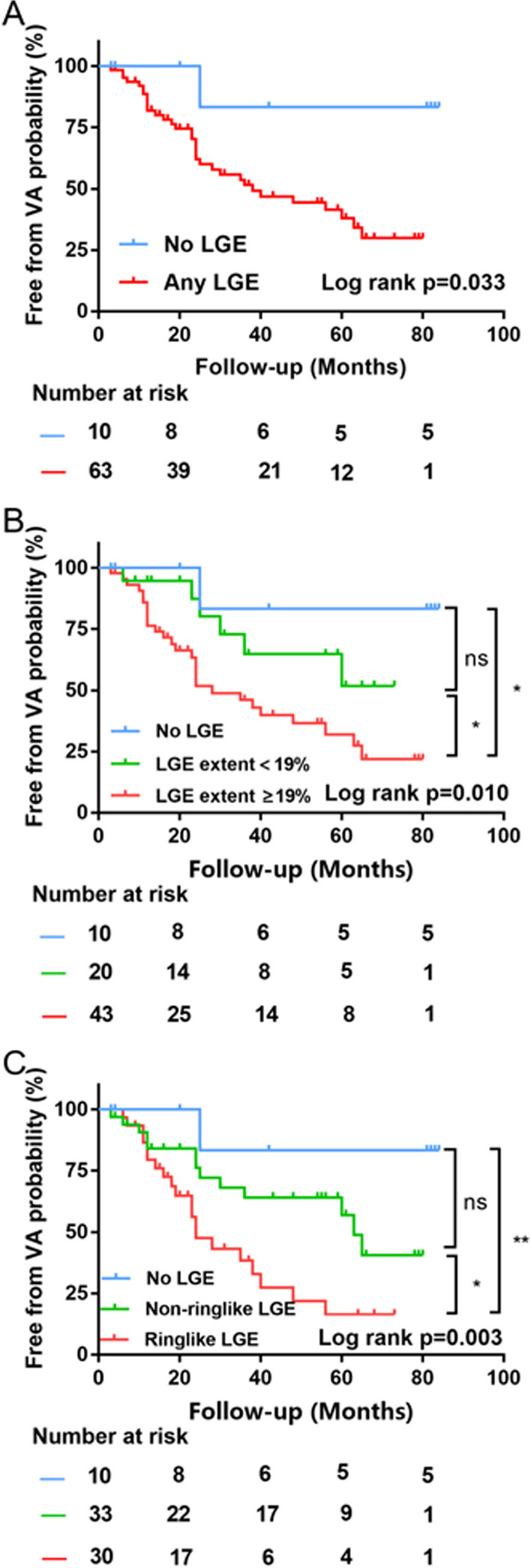
Kaplan-Meier analysis in different LGE subgroups. Kaplan–Meier survival curves showing survival free from the VA stratified by the presence (**A**), the median of LGE extent of 19% (**B**), and the patterns (**C**) of LGE. *LGE* late gadolinium enhancement, *VA* ventricular arrhythmias. Log-rank p: *p < 0.05, **p < 0.01. *ns* not statistically significant

After a median of 34 (13–56) months follow-up, sustained VA occurred in 1 of 10 (10.0%) in the no LGE group, 13 of 33 (39.4%) in the non-ringlike LGE group, and 20 of 30 (66.7%) patients in the ringlike LGE group (Table 1). The characteristics of patients with and without sustained VA events are reported in additional file (Additional file 1: Table S2). By Kaplan–Meier analysis (Fig. 5), the presence of LGE was related to a higher VA risk and the risk increased with the LGE extent. In terms of the LGE patterns, the ringlike LGE group was associated with a significantly higher risk of VA compared with the no LGE and the non-ringlike LGE groups, but no significant difference in VA risk was found between the no LGE and the non-ringlike LGE groups (Log-rank *P* = 0.112).

In the univariable Cox regression analyses (Table 3), compared with no LGE, the hazard ratios (HRs) with 95% confidence intervals (CIs) for VA were 3.31 (0.42–25.50) and 11.24 (1.51–84.37) for the non-ringlike LGE group and the ringlike LGE group, respectively. After multivariable adjustment, the association between 24-h premature ventricular contraction count (adjusted HR: 1.31, 95% CI: 0.45–3.82; *P* = 0.62), LVEF (adjusted HR: 0.97, 95% CI: 0.92–1.02; *P* = 0.19) and LGE extent (adjusted HR: 1.03, 95% CI: 0.99–1.06; *P* = 0.07) and VA event was no longer present. However, the inverted T-wave (adjusted HR: 1.21, 95% CI: 1.04–1.44; *P* = 0.006), 5-year ARVC risk score (adjusted HR: 1.05, 95% CI: 1.00–1.09; *P* = 0.034) and ringlike LGE (adjusted HR: 6.91, 95% CI: 1.89–54.60; *P* = 0.036) remained independent predictors of the occurrence of sustained VA.

**Table 3:** Univariable and multivariable cox regression analysis for predicting sustained ventricular arrhythmia

Variables	Univariable		Multivariable	
	HR (95%CI)	*P-*Value	HR (95%CI)	*P-*Value
Age (years)	1.02(1.00–1.05)	0.054		
Male, n (%)	1.48(0.73–3.00)	0.28		
BSA (m^2^)	0.43(0.08–2.50)	0.43		
Systemic hypertension, n (%)	1.05(0.47–2.34)	0.90		
Diabetes, n (%)	1.40(0.49–3.96)	0.54		
History of syncope, n (%)	1.59(0.77–3.29)	0.21		
Family history of CAD, n (%)	1.74(0.53–5.75)	0.36		
Proband, n (%)	1.99(0.96–4.09)	0.06		
NYHA III - IV, n (%)	2.00(0.98–4.08)	0.06		
NSVT, n (%)	2.00(0.96–4.20)	0.07		
24-h PVC count (≥ 1000), n (%)	2.61(1.07–6.32)	**0.034**	1.31(0.45–3.82)	0.62
Inverted T-wave, n	1.22(1.06–1.40)	**0.005**	1.21(1.04–1.44)	**0.006**
Genotype, PKP2	1.50(0.68–3.31)	0.32		
5-yr ARVC risk score, (%)	1.07(1.03–1.11)	**0.001**	1.05(1.00-1.09)	**0.034**
CMR findings				
LV EDVi (mL/m^2^)	1.01(0.98–1.03)	0.65		
LV ESVi (mL/m^2^)	1.03(1.01–1.05)	**0.047**		
LV MI (g/m^2^)	1.02(0.99–1.05)	0.16		
LV EF (%)	0.95(0.91–0.99)	**0.031**	0.97(0.92–1.02)	0.19
LV RWMA, n (%)	1.52(0.76–3.06)	0.24		
LV fat infiltration, n (%)	1.37(0.70–2.69)	0.36		
LV LGE extent (%)	1.05(1.02–1.08)	**0.013**	1.03(0.99–1.06)	0.07
LV LGE pattern, n (%)				
No LGE	Ref.		Ref.	
Non-ringlike LGE	3.31(0.42–25.50)	0.26	1.53(0.61–14.8)	0.64
Ringlike LGE	11.24(1.51–84.37)	**0.001**	6.91(1.89–54.60)	**0.036**
RV EDVi (mL/m^2^)	1.01(0.99–1.02)	0.15		
RV ESVi (mL/m^2^)	1.02(0.99–1.03)	0.11		
RV EF (%)	0.94(0.88–1.001)	0.052		
RV RWMA, n (%)	1.77(0.89–3.54)	0.11		
RV fat infiltration, n (%)	1.94(0.99–3.82)	0.054		
RV LGE, n (%)	1.82(0.91–3.64)	0.09		

Values in bold indicate P < 0.05

*HR* hazard ratio, *CI* confidence interval, *BSA* body surface area, *CAD* coronary artery disease, *NYHA* New York Heart Association, *NSVT* non-sustained ventricular tachycardia, *PVC* premature ventricular complex, *PKP2* plakophilin-2, *ARVC* arrhythmogenic right ventricular cardiomyopathy, *LV* left ventricular, *EDVi* end diastolic volume index, *ESVi* end systolic volume index, *MI* mass index, *EF* ejection fraction, *RWMA* regional wall motion abnormalities, *LGE* late gadolinium enhancement, *RV* right ventricular

### Incremental value of LVEF and LGE

**Fig. 6:**
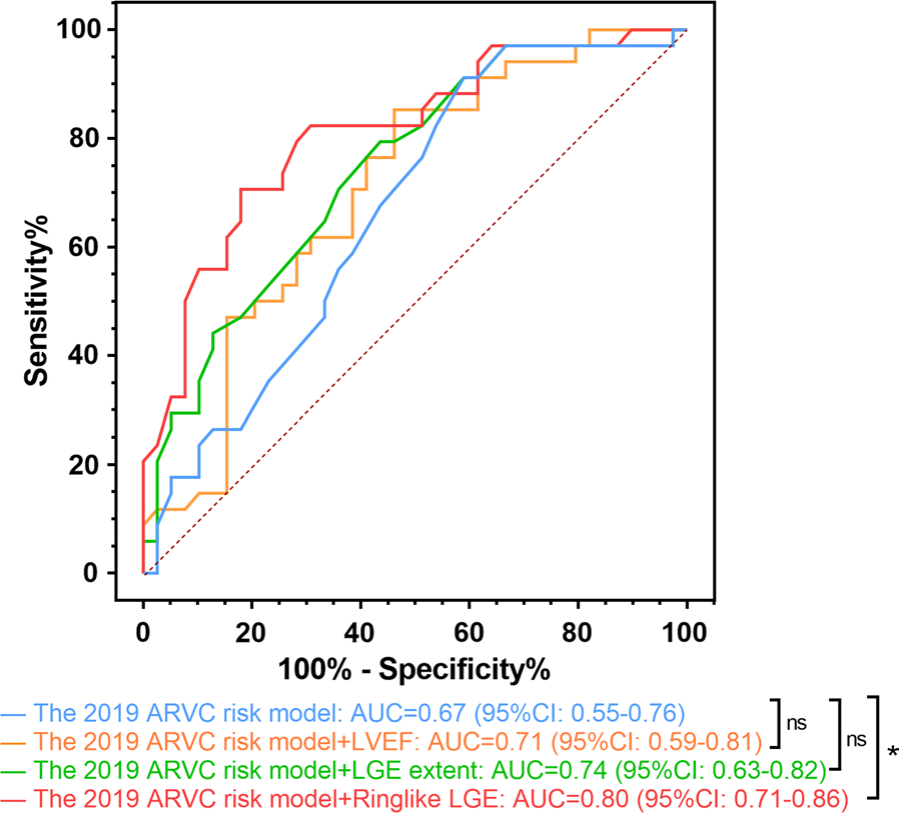
Receiver operating characteristic curve analysis for the incremental value of LVEF, LGE extent and ringlike LGE over the 2019 ARVC risk model. *LVEF* left ventricle ejection fraction, *LGE* late gadolinium enhancement, *ARVC* arrhythmogenic right ventricular cardiomyopathy, *AUC* area under curve, *CI* confidence interval. *p < 0.05. *ns* not statistically significant

Figure 6 illustrates the change in AUC when comparing the 2019 ARVC risk model separately and after adding LVEF, LGE extent and ringlike LGE pattern. The predictive performance of the 2019 ARVC risk model improved in combination with LVEF [0.67 (0.55–0.76) vs. 0.71 (0.59–0.81); *P* = 0.23] and LGE extent [0.67 (0.55–0.76) vs. 0.74 (0.63–0.82); *P* = 0.10] separately, although this did not reach statistical significance. However, when ringlike LGE was added to the risk model, the AUC significantly increased from 0.67 (95% CI: 0.55–0.76) to 0.80 (95% CI: 0.71–0.86) (*P* = 0.024).

## Discussion

In the present study, we evaluated the incremental prognosis value of ringlike LGE beyond the 2019 ARVC risk model in non-classical ACM patients. The study showed that (1) among the three patterns of LGE, the sustained VA more frequently occurred in ACM patients with the ringlike LGE pattern; (2) the presence of LGE heralded a higher risk of sustained VA over a median follow-up of 34 months. In addition, the ringlike LGE pattern was a significant independent risk predictor of sustained VA; (3) the ringlike LGE could provide incremental prognostic value for sustained VA on the basis of the 2019 ARVC risk model.

Our data demonstrated that a ringlike LGE pattern could serve as an independent predictor of VA in non-classic ACM patients. LV LGE was observed in 63 of 73 (86.3%) of patients which was higher than those in previous studies ranging from 14 to 84% [19], the possible reason may be that the majority of patients included in our study were hospitalized patients with severe CMR phenotypes. Thus, it was speculated that the high proportion of LGE (86.3%) may contribute to the relatively high event rate of sustained VA (46.6%) during the follow-up period. In accordance with the previous study [20, 21], we found that the higher extent of LGE was associated with the increased risk of VA. However, this association disappeared after multivariable adjustment in our study, and only the ringlike LGE remained an independent risk predictor for VA. This result indicated that the high VA incidence in patients with a larger LGE extent might be attributed to the ringlike LGE pattern. The discontinuity of myocardial tissue structure induced by LGE could produce conduction slowing and block predisposing for reentry circuit development [22]. Our study showed that the non-classic ACM patients with ringlike LGE pattern had more frequent occurrence of the sustained VA. In addition, this ringlike LGE pattern mainly involved the inferior and lateral segments at the subepicardial layer of LV. In general, as a substrate for arrhythmias, the pattern and location of LV LGE is closely related to the occurrence and type of cardiac arrhythmias, and its exact underlying mechanisms need further investigation.

Furthermore, this study showed that an assessment of the ringlike LGE pattern could offer valuable additional information for predicting sustained VA beyond the 2019 ARVC risk model, which seemed to underestimate the risk of patients with non-classic ACM [9, 23]. One possible reason is that ventricular function contribution in VA risk has not been appropriately evaluated yet and the RVEF impairment may be minimal or even non-existent in the non-classical subtypes, therefore interfering the predictive performance of the risk model. Indeed, our result confirmed that RVEF was not significantly different between patients with and without VA and was not associated with the presence of arrhythmic events. Conversely, LV abnormalities could provide the complementary prognostic information to the 2019 ARVC risk model, which has been confirmed to be the independent predictor of the adverse cardiovascular event [9]. However, the 2019 ARVC risk model was built on the basis of R-ACM, and a new calculator specifically for non-classical ACM should be considered. Our study confirmed that the presence of ringlike LV LGE was associated with an almost 7-fold increase in the risk of VA compared with none of LGE, which indicates the fundamental value of ringlike LGE to develop a new calculator for patients with non-classical ACM.

Notably, we did not find a significant increase in the prognostic value of LVEF after adding it to the 2019 ARVC risk model. Although a lower LVEF was observed in patients with VA than those without VA, the phenomenon may be caused by the presence of LGE. The similar conclusion was drawn in patients with dilated cardiomyopathy, with predictive models by LGE occurrence and pattern being superior to the models on the basis of LVEF [20]. In the largest sample size of ACM patients from a multinational registry, Cadrin-Tourigny et al. retrospectively demonstrated that LVEF was not a significant predictor of sustained VA [8], which was consistent with the results of this study. However, several European groups have proved a significant independent association of reduced LV function with SCD or life-threatening arrhythmic events [24, 25], and the presence of LV dysfunction has an incremental power in predicting the long-term adverse outcomes compared with RV dysfunction alone [24]. Differences in genes and populations enrolled from different centers may be driving these disparate findings with a higher prevalence of patients with DSP mutations in the European cohorts [26]. Nevertheless, this reflects real-world clinical practice.

The presence of ringlike LGE pattern may be related to DSP mutation. In this study, the majority of DSP mutations [6 of 8 (75.0%)] were observed in patients with ringlike LGE pattern. A study about molecular mechanisms has shown that the desmosomal DSP mutation was more likely to occur in patients with LD-ACM compared with other desmosomal gene mutations such as PKP2 [27]. The ringlike LGE on the subepicardial or midmyocardial layer may be the most representative characteristic in the diagnosis of LD-ACM [28]. These findings suggested that there might be a potential relationship between ringlike LGE pattern and DSP mutation. In a genotype-imaging phenotype study, Augusto et al. demonstrated that ringlike LGE was observed in 78% of DSP genotypes, which showed a favorable suggestive value of ringlike LGE for DSP genotype [18]. Given that the limited genetic sample size in our study, caution should be mentioned when extrapolating genetic association in terms of the LV LGE pattern.

### Limitations

Our study had several limitations. First, although the sample size of the current study was limited based on the strict diagnostic criteria, the calculated statistical power was 82%, which demonstrated the creditability of our study results. Of course, future studies with a larger sample size are needed to verify our conclusions. Second, we should note the limitation that the 2019 ARVC risk model was intended to be used for R-ACM patients. LV imaging biomarkers such as the ringlike LGE should be considered to develop a new calculator specifically for non-classical ACM patients. Third, the Padua criteria were published in 2020, and its accuracy in diagnosing ACM has not been well validated at present, but it is the relatively appropriate way to diagnose non-classical ACM. Finally, we did not evaluate the prognostic role of T1 mapping in this study, because at the time of enrollment, the mapping technique was unavailable in our center.

## Conclusion

In conclusion, the presence of LV LGE is associated with sustained VA, and the presence of ringlike LGE is an independent predictor of sustained VA in non-classical ACM patients. Importantly, ringlike LGE might provide incremental prognostic information for sustained VA beyond the 2019 ARVC risk model. Further investigations are needed to evaluate the impact of ringlike LGE on decision-making and whether these decisions benefit patients.

### Supplementary Information

**Additional file 1: Table S1.** Interobserver reproducibility in LV LGE pattern classification. **Table S2.** Clinical characteristics and imaging findings according to the presence of sustained ventricular arrhythmia. **Figure S1.** Schematic illustration and corresponding CMR imaging of three LGE patterns. **A** No LGE; **B**-**D** non-ringlike LGE: there were less than three contiguous segments with LGE in the same short-axis slice; **E**, **F** ringlike LGE: there were full involvement of at least three contiguous segments with LGE at the subepicardial or midmyocardial layer in the same short-axis slice. *CMR* cardiac magnetic resonance; *LGE* late gadolinium enhancement.

## Data Availability

The datasets acquired and/or analyzed during the current study are available from the corresponding author on reasonable request.

## Abbreviations

ACM: Arrhythmogenic cardiomyopathy
VA: Ventricular arrhythmias
SCD: Sudden cardiac death
ARVC: Arrhythmogenic right ventricular cardiomyopathy
CMR: Cardiac magnetic resonance
ICD: Implantable cardioverter-defibrillator
LV: Left ventricular
LGE: Late gadolinium enhancement
DCM: Dilated cardiomyopathy
HR: Hazard ratio
CI: Confidence interval
AUC: Area under curve
