# Supplementary material for: Ringlike late gadolinium enhancement provides incremental prognostic value in non-classical arrhythmogenic cardiomyopathy
Source: J Cardiovasc Magn Reson. 2023 Nov 30;25:72. doi: 10.1186/s12968-023-00986-1 (PMC10687920; doi:10.1186/s12968-023-00986-1)
Supplement: Supplementary file 1 — Additional file 1: Table S1. Interobserver reproducibility in LV LGE pattern classification. Table S2. Clinical characteristics and imaging findings according to the presence of sustained ventricular arrhythmia. Figure S1. Schematic illustration and corresponding CMR imaging of three LGE patterns. A No LGE; B-D non-ringlike LGE: there were less than three contiguous segments with LGE in the same short-axis slice; E, F ringlike LGE: there were full involvement of at least three contiguous segments with LGE at the subepicardial or midmyocardial layer in the same short-axis slice. CMR cardiac magnetic resonance; LGE late gadolinium enhancement. [file 12968_2023_986_MOESM1_ESM.docx]

**Additional file**

**Diagnostic criteria**

**“Padua criteria” for diagnosis of Arrhythmogenic Cardiomyopathy**

| **Category** | **Right ventricle (upgraded 2010 ITF diagnostic criteria)** | **Left ventricle (new diagnostic criteria)** |
| --- | --- | --- |
| I. Morpho-functional  ventricular  abnormalities | By echocardiography, CMR or angiography:  Major  • Regional RV akinesia, dyskinesia, or bulging plus one of the following:  - global RV dilatation (increase of RV EDV according to the imaging test  specific nomograms)  - global RV systolic dysfunction (reduction of RV EF according to the  imaging test specific nomograms)  Minor  • Regional RV akinesia, dyskinesia or aneurysm of RV free wall | By echocardiography, CMR or angiography:  Minor  • Global LV systolic dysfunction (depression of LV EF or reduction of echocardiographic global longitudinal strain), with or without LV dilatation (increase of LV EDV according to the imaging test specific nomograms for age, sex, and BSA)  Minor  • Regional LV hypokinesia or akinesia of LV free wall, septum, or both |
| II. Structural  myocardial  abnormalities | By CE-CMR: Major  • Transmural LGE (stria pattern) of  ≥1 RV region(s) (inlet, outlet, and apex in 2 orthogonal views)  By EMB (limited indications): Major  • Fibrous replacement of the myocardium in ≥1 sample, with or without fatty tissue | By CE-CMR: Major  • LV LGE (stria pattern) of ≥1 Bull's Eye segment(s) (in 2 orthogonal views) of the free wall (subepicardial or midmyocardial), septum, or both (excluding septal junctional LGE) |
| III. Repolarization  abnormalities | Major  • Inverted T waves in right precordial leads (V1,V2, and V3) or beyond in  individuals with complete pubertal development (in the absence of  complete RBBB)  Minor  • Inverted T waves in leads V1 and V2 in individuals with completed pubertal development (in the absence of complete RBBB)  • Inverted T waves in V1,V2,V3 and V4 in individuals with completed pubertal development in the presence of complete RBBB | Minor  • Inverted T waves in left precordial leads (V4-V6) (in the absence of complete LBBB) |
| IV. Depolarization  abnormalities | Minor  • Epsilon wave (reproducible low-amplitude signals between end of QRS  complex to onset of the T wave) in the right precordial leads (V1 to V3)  • Terminal activation duration of QRS ≥ 55 ms measured from the nadir of the S wave to the end of the QRS, including R', in V1, V2, or V3 (in the absence of complete RBBB) | Minor  • Low QRS voltages (< 0.5 mV peak to peak) in limb leads (in the absence of obesity, emphysema, or pericardial effusion) |
| V. Ventricular  arrhythmias | Major  • Frequent ventricular extrasystoles (> 500 per 24 h), non-sustained or sustained ventricular tachycardia of LBBB morphology  Minor  • Frequent ventricular extrasystoles (>500 per 24 h), non-sustained or sustained ventricular tachycardia of LBBB morphology with inferior axis (“RVOT pattern”) | Minor  • Frequent ventricular extrasystoles (>500 per 24 h), non-sustained or sustained ventricular tachycardia with a RBBB morphology (excluding the “fascicular pattern”) |
| VI. Family  history/genetics | Major  • ACM confirmed in a first-degree relative who meets diagnostic criteria  • ACM confirmed pathologically at autopsy or surgery in a first degree relative  • Identification of a pathogenic or likely pathogenetic ACM mutation in the patient under evaluation  Minor  • History of ACM in a first-degree relative in whom it is not possible or practical to determine whether the family member meets diagnostic criteria  • Premature sudden death (<35 years of age) due to suspected ACM in a first-degree relative  • ACM confirmed pathologically or by diagnostic criteria in a second-degree relative | |


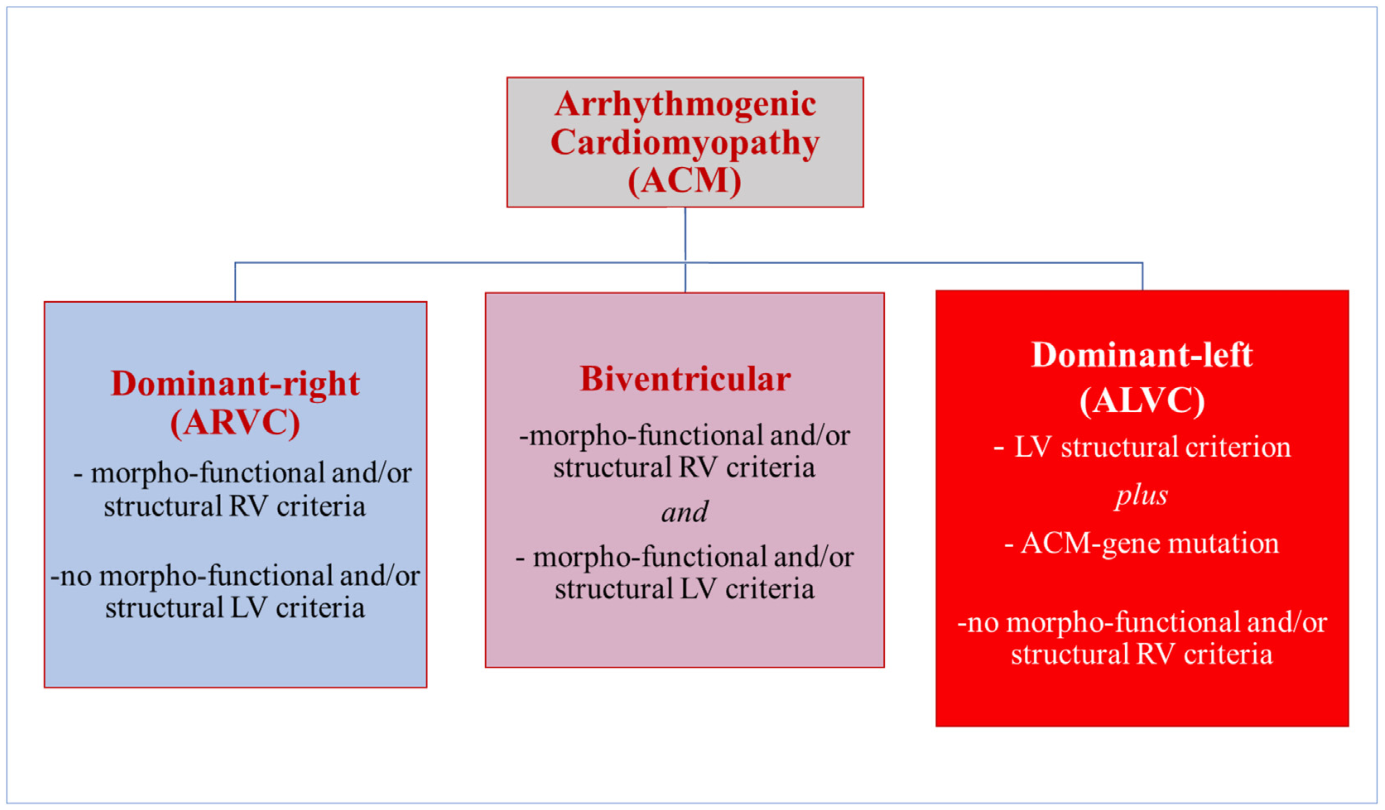


**Diagnosis of phenotypic variants of ACM in patients fulfilling the Padua criteria.**

Supplemental diagnostic criteria above was cited from Corrado et al [1].

According to the Supplemental diagnostic criteria above, biventricular arrhythmogenic cardiomyopathy (Bi-ACM) can be diagnosed in those patients meeting ≥ 1 morpho-functional and/or structural abnormalities of both the RV and LV (i.e., patients fulfilling RV and LV phenotypic criteria). Left dominant arrhythmogenic cardiomyopathy (LD-ACM) can be diagnosed in patients who showed structural LV abnormalities (with or without morpho-functional alterations), with the demonstration of an ACM-causing gene-mutation, in the absence of RV abnormalities.

*ACM* arrhythmogenic cardiomyopathy, *BSA* body surface area, *EDV* end diastolic volume, *EF* ejection fraction, *ITF* International Task Force, *LBBB* left bundle-branch block, *LGE* late gadolinium enhancement, *LV* left ventricle, *RBBB* right bundle-branch block, *RV* right ventricle, *RVOT* right ventricular outflow tract

**Methods**

**CMR Protocol**

The T2-weighted turbo spin echo sequences with and without fat suppression were performed to obtain T2-weighted images, comprising of a stack of short-axis planes covering the entire LV from the apex to the base, and axis planes centered on the left and right ventricle. The parameters of T2-weighted images were as the following: field of view (FOV), 230×230 mm^2^; voxels, 2×2×8 mm^3^; repetition time (TR) /echo time (TE), 1500/60 ms; interslice gap = 2 mm; and flip angle, 90°.

The single-shot balanced steady-state free precession (bSSFP) sequences with breath-hold were performed to obtain cine images, comprising of the axial plane, a stack of short-axis planes covering the entire LV from the apex to the base, and long-axis planes (two-, three-, and four-chamber views). The parameters of cine image were as the following: FOV, 230×230 mm^2^; voxels, 2×2×8 mm^3^; TR/TE, shortest/shortest; sense factor, 2; interslice gap = 2 mm; and flip angle, 45°.

LGE imaging was acquired 10-15 min after intravenous administration of Gd-based contrast agent (Magnevist, Bayer HealthCare Pharmaceuticals) with a dosage of 0.2 mmol/kg in the same axial plane, short-axis and long-axis views using a phase-sensitive inversion-recovery sequence. The parameters were as follows: FOV, 230×230 mm^2^; voxels, 2×2×8 mm^3^; TR/TE, shortest/ shortest; sense factor, 2; interslice gap = 2 mm; flip angle, 25°; and TI, measured at that time to null the signal from the normal myocardium.

**CMR Analysis**

The endocardial and epicardial contours of the left ventricle (papillary muscles were excluded) and the endocardial contour of the right ventricle were drawn manually on short-axis cine images at end-diastole and end-systole for functional and structural evaluation parameters, including biventricular ejection fraction (EF), end-diastole volume (EDV), end-systolic volume (ESV), and LV myocardial mass, with indexing for body surface area.

The LGE was visually assessed for each segment using a modified American Heart Association (AHA) 17-segment left ventricular (LV) model and deemed to be positive if there was a region of ascertainable high signal intensity on both short- and orthogonal long-axis LGE images [2, 3]. Quantification of LGE extent was performed in the short-axis slices by manually drawing endocardial and epicardial borders and selecting a region of interest (ROI) in the remote healthy myocardium. Mean signal intensity and standard deviations (SD) of the ROI were measured, and enhanced myocardium was defined as a myocardium with a signal intensity ≥ 5 SDs above the mean of the ROI [4]. The extent of LGE was expressed as a percentage of the LV mass, which was derived by dividing the LGE mass by the myocardial mass.

The pattern of LGE was independently assessed by two experienced investigators (YL.Y. and XY. W. with 10 years of CMR post-possessing experience) who were blinded to the patients’ clinical data. When investigators disagreed, the decision was adjudicated by a third investigator (H.L with 30 years of CMR post-possessing experience). Reproducibility analysis of inter-observer for the pattern of LGE was evaluated by Kappa-statistics.

**References**

1. Corrado D, Perazzolo Marra M, Zorzi A, Beffagna G, Cipriani A, Lazzari M, et al. Diagnosis of arrhythmogenic cardiomyopathy: The Padua criteria. Int J Cardiol. 2020;319:106-14.

2. Cerqueira M, Weissman N, Dilsizian V, Jacobs A, Kaul S, Laskey W, et al. Standardized myocardial segmentation and nomenclature for tomographic imaging of the heart. A statement for healthcare professionals from the Cardiac Imaging Committee of the Council on Clinical Cardiology of the American Heart Association. Circulation. 2002;105:539-42.

3. Rubis P, Dziewiecka E, Szymanska M, Banys R, Urbanczyk-Zawadzka M, Krupinski M, et al. Lack of relationship between fibrosis-related biomarkers and cardiac magnetic resonance-assessed replacement and interstitial fibrosis in dilated cardiomyopathy. Cells. 2021;10:1295.

4. Bondarenko O, Beek AM, Hofman MB, Kuhl HP, Twisk JW, van Dockum WG, et al. Standardizing the definition of hyperenhancement in the quantitative assessment of infarct size and myocardial viability using delayed contrast-enhanced CMR. J Cardiovasc Magn Reson. 2005;7:481-5.

**Table S1 Interobserver reproducibility in LV LGE pattern classification**

|  |  | **Observer 1** | | |
| --- | --- | --- | --- | --- |
|  | LGE patterns | Ringlike LGE  (n=30) | Non-ringlike LGE  (n=33) | No LGE  (n=10) |
| **Observer 2** | Ringlike LGE  (n=32) | 29 | 3 | 0 |
|  | Non-ringlike LGE  (n=31) | 1 | 30 | 0 |
|  | No LGE  (n=10) | 0 | 0 | 10 |

Classification of the patterns of LGE was assessed by two operators and showed excellent interobserver reproducibility (Kappa = 0.91, *P* < 0.001).

*LV* left ventricular, *LGE* late gadolinium enhancement

**Table S2 Clinical characteristics and imaging findings according to the presence of sustained ventricular arrhythmia**

| **Variables** | **Sustained VA** | | ***P-*Value** |
| --- | --- | --- | --- |
|  | **Yes (n=34)** | **No (n=39)** |  |
| Clinical characteristics |  |  |  |
| Age (years) | 43.6±12.5 | 35.6±15.0 | **0.017** |
| Male, n (%) | 26(76.5) | 25(64.1) | 0.25 |
| BSA (m^2^) | 1.6±0.2 | 1.7±0.2 | 0.61 |
| Systemic hypertension, n (%) | 8(23.5) | 5(12.8) | 0.23 |
| Diabetes, n (%) | 4(11.8) | 3(7.7) | 0.85 |
| History of syncope, n (%) | 11(32.4) | 4(10.3) | **0.041** |
| Family history of CAD, n (%) | 3(8.8) | 2(5.1) | 0.87 |
| Proband, n (%) | 23(67.6) | 16(41.0) | **0.023** |
| NYHA III - IV, n (%) | 12(35.3) | 8(20.5) | 0.16 |
| NSVT, n (%) | 24(70.6) | 17(43.6) | **0.020** |
| 24-h PVC count (≥1000), n (%) | 28(82.4) | 21(53.8) | **0.010** |
| Inverted T-wave, n | 3(1-5) | 0(0-3) | **0.002** |
| Genotype (n=32) | 18(52.9) | 14(35.9) | 0.14 |
| PKP2 | 8(23.5) | 7(17.9) |  |
| DSP | 6(17.6) | 2(5.1) |  |
| DSG2 | 2(5.9) | 2(5.1) |  |
| DSC2 | 1(2.9) | 1(2.6) |  |
| Other | 1(2.9) | 2(5.1) |  |
| Therapy, n (%) |  |  |  |
| Beta-blockers | 29(85.3) | 30(76.9) | 0.37 |
| ACE inhibitors | 16(47.1) | 15(38.5) | 0.46 |
| Antiarrhythmic drug | 22(64.7) | 16(41.0) | **0.043** |
| Diuretic agent | 12(35.3) | 7(17.9) | 0.09 |
| ICD | 18(52.9) | 9(23.1) | **0.008** |
| 5-yr ARVC risk score, (%) | 21.4±7.5 | 16.7±8.3 | **0.014** |
| CMR findings |  |  |  |
| LV EDVi (mL/m^2^) | 96±15 | 97±17 | 0.79 |
| LV ESVi (mL/m^2^) | 57±18 | 54±17 | 0.43 |
| LV MI (g/m^2^) | 69±13 | 66±12 | 0.31 |
| LV EF (%) | 42±7.4 | 47±8.4 | **0.031** |
| LV RWMA, n (%) | 13(38.2) | 14(35.9) | 0.84 |
| LV fat infiltration, n (%) | 18(52.9) | 13(33.3) | 0.09 |
| LGE extent (%) | 24(16-32) | 14(0-20) | **<0.001** |
| LV LGE pattern, n (%) |  |  |  |
| No LGE | 1(2.9) | 9(23.1) | **0.031** |
| Non-ringlike LGE | 13(38.2) | 20(51.3) | 0.26 |
| Ringlike LGE | 20(58.8) | 10(25.6) | **0.004** |
| RV EDVi (mL/m^2^) | 104±23 | 96±29 | 0.24 |
| RV ESVi (mL/m^2^) | 50±12 | 46±18 | 0.22 |
| RV EF (%) | 51±5.3 | 53±5.7 | 0.16 |
| RV RWMA, n (%) | 15(44.1) | 16(41.0) | 0.79 |
| RV fat infiltration, n (%) | 17(50.0) | 12(30.8) | 0.09 |
| RV LGE, n (%) | 21(61.8) | 15(38.5) | **0.047** |

Continuous variables are presented as mean ± standard deviation or median (interquartile range) and discrete variables as n (%).

Values in bold indicate P < 0.05.

Abbreviations: same as Table S1. *VA* ventricular arrhythmia, *BSA* body surface area, *CAD* coronary artery disease, *NYHA* New York Heart Association, *NSVT* non-sustained ventricular tachycardia, *PVC* premature ventricular complex, *PKP2* plakophilin-2, *DSP* desmoplakin, *DSG2* desmoglein-2, *DSC2* desmocollin-2, *ACE* angiotensin-converting enzyme, *ICD* implantable cardioverter-defibrillator, *ARVC* arrhythmogenic right ventricular cardiomyopathy, *EDVi* end diastolic volume index, *ESVi* end systolic volume index, *MI* mass index, *EF* ejection fraction, *RWMA* regional wall motion abnormalities, *RV* right ventricular


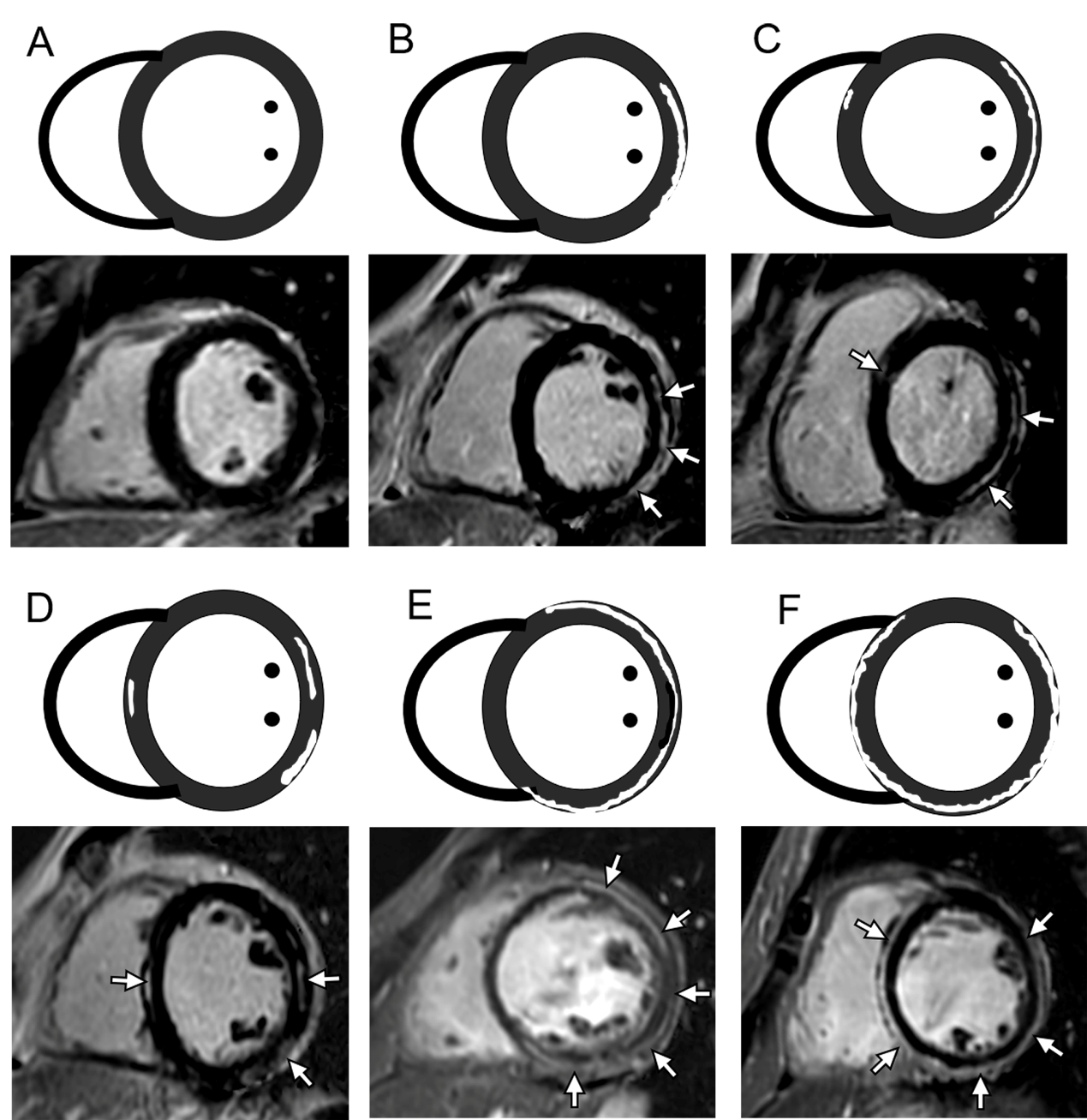


**Figure S1** **Schematic illustration and corresponding CMR imaging of three LGE patterns.**

**A** No LGE; **B-D** non-ringlike LGE: there were less than three contiguous segments with LGE in the same short-axis slice; **E, F** ringlike LGE: there were full involvement of at least three contiguous segments with LGE at the subepicardial or midmyocardial layer in the same short-axis slice.

*CMR* cardiac magnetic resonance, *LGE* late gadolinium enhancement
